# Low Vitamin D Concentration Is Not Associated with Increased Mortality and Morbidity after Cardiac Surgery

**DOI:** 10.1371/journal.pone.0063831

**Published:** 2013-05-28

**Authors:** Alparslan Turan, Martin Grady, Jing You, Edward J. Mascha, Worasak Keeyapaj, Ryu Komatsu, C. Allen Bashour, Daniel I. Sessler, Leif Saager, Andrea Kurz

**Affiliations:** 1 Department of Outcomes Research, Cleveland Clinic, Cleveland, Ohio, United States of America; 2 Department of Regional Anesthesiology, Cleveland Clinic, Cleveland, Ohio, United States of America; 3 Department of Quantitative Health Sciences, Cleveland Clinic, Cleveland, Ohio, United States of America; 4 Department of Cardiothoracic Anesthesiology, Cleveland Clinic, Cleveland, Ohio, United States of America; 5 Anesthesiology Institute, Cleveland Clinic, Cleveland, Ohio, United States of America; 6 Population Health Research Institute, McMaster University, Hamilton, Ontario, Canada; Università Vita-Salute San Raffaele, Italy

## Abstract

**Objective:**

To determine the effect of vitamin D on postoperative outcomes in cardiac surgical patients.

**Design:**

Retrospective study.

**Setting:**

Single institution-teaching hospital.

**Participants:**

Adult cardiac surgical patients with perioperative 25-hydroxyvitamin D measurements.

**Interventions:**

None. We gathered information from the Cardiac Anesthesiology Registry that was obtained at the time of the patients’ visit/hospitalization.

**Measurements and Main Results:**

We used data of 18,064 patients from the Cardiac Anesthesiology Registry; 426 patients with 25-hydroxyvitamin D measurements met our inclusion criteria. Association with Vitamin D concentration and composite of 11 cardiac morbidities was done by multivariate (i.e., multiple outcomes per subject) analysis. For other outcomes separate multivariable logistic regressions and adjusting for the potential confounders was used. The observed median vitamin D concentration was 19 [Q1-Q3∶12, 30] ng/mL. Vitamin D concentration was not associated with our primary composite of serious cardiac morbidities (odds ratio [OR], 0.96; 95% CI, 0.86–1.07). Vitamin D concentration was also not associated with any of the secondary outcomes: neurologic morbidity (P = 0.27), surgical (P = 0.26) or systemic infections (P = 0.58), 30-day mortality (P = 0.55), or length of initial intensive care unit (ICU) stay (P = 0.04).

**Conclusions:**

Our analysis suggests that perioperative vitamin D concentration is not associated with clinically important outcomes, likely because the outcomes are overwhelmingly determined by other baseline and surgical factors.

## Introduction

Heart disease is the leading cause of death in the United States and most developed countries. As a consequence, over a million cardiac surgeries are performed every year [1]. Recovery after cardiac surgery is frequently complicated by arrhythmias, low cardiac output, heart block, heart failure, and pulmonary edema [2]. These serious complications prolong hospitalization and markedly increase cost in afflicted patients; preventing them would thus reduce morbidity and, presumably, mortality – to say nothing of the cost of care.

Vitamin D deficiency is an alarming global health problem. It has been estimated that one billion people worldwide have vitamin D deficiency or insufficiency [3]. Inadequate serum vitamin D concentrations have been observed in up to 90% of some young and apparently healthy adult populations [4]. The non-traditional role of vitamin D has been extensively investigated in recent years [5], [6]. Vitamin D deficiency is prevalent in patients with cardiovascular diseases such as coronary, peripheral arterial disease, and heart failure – and is strongly associated with increased cardiovascular risk and events [7], [8], [9], [10], [11]. In a recent study, for example, Vitamin D deficiency was found in almost all of the patients who presented with acute myocardial infarction [12]. Despite reports on the prevalence of hypo-vitaminosis D in the general population and significant worsening of cardiovascular outcomes with vitamin D deficiency, there is a paucity of studies focusing on surgical patients.

Beside its traditional role in bone maintenance, vitamin D level has been linked to several factors that might influence outcomes after cardiac surgery. Vitamin D not only has cardio-protective effects, but is also neuroprotective. In an animal model, pre-treatment with vitamin D significantly reduced the brain infarct size and inadequate vitamin D was associated with neuronal vulnerability [13], [14]. Vitamin D also has an important linkage to both innate and acquired immune systems through the production of antimicrobial peptides-particularly cathelicidin [3], [15]. Furthermore, serum vitamin D might play a significant role in lower respiratory tract infections and immune response modulation. Low serum vitamin D concentrations are correlated with severity of acute lower respiratory tract infections [16] and intestinal Vitamin D system plays a critical role in maintaining both mucosal immunity and epithelial cell growth [17]. Thus vitamin D seems to play an important role in infection prevention. But whether vitamin D contributes to development of perioperative infections remains unknown.

There are thus compelling reasons to believe that low perioperative vitamin D concentrations may increase cardiac morbidity, neurologic complications, and infections after cardiac surgery. Specifically, we tested the *primary hypothesis* that patients with lower perioperative vitamin D concentrations have higher risk of serious cardiac morbidities after adult cardiac surgery. Our *secondary hypotheses* were that patients with lower perioperative vitamin D concentrations have higher risk of 30-day postoperative mortality, neurologic morbidity, surgical and systemic infectious, and a prolonged duration of hospitalization.

## Methods

With approval and waiver of consent from the Cleveland Clinic Institutional Review Board, patient information was obtained from the Cardiac Anesthesiology registry. Data were prospectively collected in a standardized fashion according to strict definitions of preoperative characteristics, intraoperative information, and postoperative outcomes from medical records and physical assessment, anesthesia records, and clinical care notes (Appendix S1). Clinical information was collected at the patient’s bedside in the cardiovascular ICU following surgery. Supplemental demographic and clinical data available in the Cleveland Clinic perioperative health documentation system were imported into the registry though manual and mechanized interfaces. All data were collected daily by experienced and specially trained research personnel in a prospective manner concurrent with patient care. Data validations were built into the registry to ensure data quality. Additional mechanized validations were performed quarterly to identify any quality issues that may not have been identified by the built-in validations.

In this study all patients who had any 25-hydroxyvitamin D measurement between 3 months before surgery until 1 month after were considered for inclusion. A single Vitamin D concentration value was used for the analysis; if multiple measurements were obtained within the above time frame, the value measured closest to the date of surgery was used. Vitamin D concentrations were obtained from the Laboratory Medicine registry. We excluded pediatric patients, patients with American Society of Anesthesiologists Physical Status greater than 4, and patients who did not have general anesthesia.

### Primary Analysis

The primary outcome was a set of 11 cardiac morbidities, including use of an intra-aortic balloon pump (IABP), asystole, requirement for extra corporeal membrane oxygenator (ECMO), open chest in the intensive care (whether unable to close or opened in the unit), ventricular tachycardia or fibrillation, atrial arrhythmia, low cardiac output (CO), permanent pacer, heart block, need for cardioversion, and pulmonary edema (Appendix S2).

Given that the incidences of the individual cardiac morbidities vary considerably (from 0.9% to 30.3%), analyzing the set of cardiac morbidities as a collapsed composite of “any-versus-none” using the usual logistic regression analysis would give results, which were “driven” by one or more morbidities with high incidences. Furthermore, the individual morbidities would not likely be considered by researchers or patients to have exactly the same severity, which cannot be accounted for the “any-versus-none” approach. We therefore employed a multivariate (i.e., one record/outcome/patient) analysis to simultaneously capture information on individual morbidities for a patient, the correlations among morbidities, and allow severity weighting of the morbidities.

Specifically, we estimated the average relative effect of vitamin D concentration across the 11 individual cardiac morbidities using a specific multivariate generalized estimating equation (GEE) model which averages the log-odds ratios across the cardiac morbidities in a “distinct effects” model [18], [19], using an unstructured covariance matrix across the morbidities. The average relative effect method is preferred over the more standard common effect GEE odds ratio when incidences of the individual components vary non-trivially, as in our study [18]. We also weighted each morbidity by a clinical severity weight estimated as the median score for that morbidity (from 1 to 100, 100 being most severe) across evaluations by nine independent anesthesiologists who were otherwise uninvolved in this study (Appendix S2).

We distinguished potential confounders (i.e., variables potentially effecting both vitamin D concentration and outcome) from ‘mediator variables’ (i.e., variables deemed to potentially lie on the causal pathway between vitamin D concentration and outcome, such as, congestive heart failure which might be caused by vitamin D deficiency and thus mediate the effect of vitamin D deficiency on the outcome). Specifically, age, gender, race, body mass index, smoking status, dialysis, and ETOH were considered as potential confounders. The following 13 conditions were deemed *a priori* to potentially mediate part of the effect of vitamin D deficiency on outcome: congestive heart failure, hypertension, vascular surgery or dilatations, vascular heart disease, carotid surgery, carotid disease, stroke, atrial fibrillation, atrial flutter, ventricular tachycardia, ventricular fibrillation, junctional rhythmus, and myocardial infarction.

Models were fit both 1) only adjusting for the potential confounding variables but not mediator variables in order to estimate the “total” effect of vitamin D concentration through the 13 available mediator variables listed above and any other unmeasured mediator variables, to the extent possible in a non-randomized study, and 2) adjusting for confounding variables as well as the *a-priori* specified mediator covariables to estimate the “direct” effect of vitamin D concentration by isolating the independent effect of vitamin D concentration on outcome in presence of the specified mediator variables.

We assessed the sensitivity of our main results to choice of methods by estimating the total effect of vitamin D concentration on the set of outcomes while ignoring the clinical severity weights and also by using the more standard common effect GEE odds ratio which gives more weight to higher incident outcome components.

Secondarily, we assessed the heterogeneity of the Vitamin D concentration effect across the individual cardiac morbidities in a separate “distinct-effects” GEE model in which the odds ratios of individual morbidities were compared [19]. Significant heterogeneity, especially in opposite directions, would suggest that the individual odds ratios be given more importance than the overall odds ratio. Since heterogeneity was found (vitamin D concentration -by-outcome interaction, P<0.001), and estimated odds ratios were in both directions, we reported the 11 individual odds ratios from the distinct effects GEE model alone, adjusting for the same set of potential confounders as above. A Bonferroni correction for multiple comparisons was employed to control the Type I error at 0.05, so that P<0.0045 was considered significant (i.e., 0.05/11 = 0.0045).

### Secondary Analyses

The secondary outcomes were neurologic morbidity (including focal and global deficits), surgical infection (including empyema, endocarditis, mediastinitis, Sternal Wound infection, and wound), systemic infection (including bacteremia, fungemia, line sepsis, sepsis syndrome, and septic shock), 30-day mortality, initial intensive care unit (ICU) length of stay (LOS), respiratory morbidity (including pneumonia, ARDS, aspiration, pneumonia, atelectasis, Bronchospasms, respiratory insufficient/distress, and respiratory failure), and use of vasopressor on day of surgery or postoperative day 1. All the outcomes were postoperative 30-day outcomes (Appendix S3).

We assessed the relationships between vitamin D concentration and each of the following binary secondary outcomes (including neurologic morbidity, surgical and systemic infections, and 30-day mortality) using separate multivariable logistic regression models and adjusting for the potential confounders. We assessed the relationship between vitamin D concentration and initial ICU LOS by a Cox proportional hazards regression adjusting for potential confounders. The response variable was discharged alive (yes/no), and patients who died during ICU stay were analyzed as never being discharged alive by assigning a follow-up time one day longer than the longest observed discharged alive time. A Bonferroni correction was used to adjust for the multiple testing. Thus, 99% confidence intervals (CI) were reported; and the significance criterion for the five secondary outcomes was P<0.01 (i.e., 0.05/5). Finally, we summarized the incidences of respiratory morbidity and use of vasopressors.

### Sample Size Considerations

Available power for the study was assessed by comparing low versus normal concentrations of vitamin D (>20 and ≤20 ng/mL) on the set of cardiac morbidities using a SAS macro developed for designs with multiple binary correlated endpoints (“multibinpow”) [20] using 1,000 simulations. We assumed that for patients with vitamin D concentration ≤20 ng/mL the incidences of the individual cardiac morbidities were 5% for IABP, asystole, ECMO, and open chest; 10% for permanent pacer, heart block, and cardioversion; 15% for pulmonary edema and VT/VF; and 30% for atrial arrhythmia and low CO, respectively. With 400 patients (200/group), we had more than 80% power at the 0.05 significance level to detect an average relative effect odds ratio of 0.7 or less for patients with vitamin D concentration >20 vs. ≤20 ng/mL, assuming a compound symmetric correlation structure with a between-outcome correlation of 0.05. In fact, our study had higher power because the vitamin D concentration was analyzed as a continuous exposure.

SAS version 9.3 (SAS Institute, Cary, NC, USA) and R version 2.12.0 (The R Foundation for Statistical Computing, Vienna, Austria) were used for all statistical analysis.

## Results

We used data of 18,064 patients from the Cardiac Anesthesiology Registry that was acquired between 2006 and 2010. Among these patients, 493 patients had a 25-hydroxyvitamin D measurement between 3 months prior to and 1 month following cardiac surgery. Patients who did not undergo general anesthesia, or with an American Society of Anesthesiologists physical status of 5 or more, or younger than 18 were excluded (441 remaining); patients with any missing potential confounders or mediator variables were further excluded, leaving 426 unique patients available for analysis. These patients, on average, were younger, sicker (higher ASA physical status), and more likely to have lower hematocrit and albumin, to have higher blood urea nitrogen, creatinine, and bilirubin, to have myocardial infraction, diabetes, cardio shock, congestive heart failure, dysrhythmias, atrial fibrillation, and ventricular tachycardia and fibrillation as compared to the remaining 17,638 cardiac surgical patients who were not analyzed (absolute standardized difference ≥0.20; Appendix S4). In addition, we propensity score matched our study patients to the remaining cardiac surgical patients on all the available baseline variables and exact matched on type of procedure. We found that our study patients were 1.64 times (95% CI: 1.19, 2.25) more likely to experience cardiac morbidity than the remaining cardiac surgery patients, indicating that our study patients may more likely to have comorbidities other than those listed above.

Among the 426 patients, the observed median vitamin D concentration was 19 [Q1–Q3∶12, 30] ng/mL. Appendix S5 shows the summary statistics of baseline and intra-operative characteristics by quartiles of the serum vitamin D concentration. The median [Q1, Q3] difference between the vitamin D concentration observation date and the date of surgery was 5 [−3, 22] days (i.e., a median 5 days before surgery).

### Primary Results

Vitamin D concentration was not associated with our primary set of serious cardiac morbidities, either adjusting only for potential confounding variables (model 1, P = 0.46) or after adjusting for both potential confounders and mediator variables (model 2, P = 0.87). The corresponding estimated severity-weighted average relative effect odds ratios across the 11 individual morbidities were 0.96 (95% CI: 0.86, 1.07) and 1.01 (0.90, 1.13) for a 5-unit increase in vitamin D concentration for models 1 and 2, respectively (Table 1). In model 1, the estimated odds ratio assesses the overall association (‘total’ effect) between vitamin D concentration and outcome, including the effects through the expected mediator variables and any unmeasured variables, whereas model 2 estimates the ‘direct’ effect of vitamin D concentration after removing the effects of the mediator variables on the outcome.

**Table 1:** Severity-adjusted* average relative effect of vitamin D concentration across 11 cardiac morbidities among 426 cardiac surgical patients.

Model adjustment	OR# (95% CI)	P
1. Potential confounders¶ - *‘Total’ effect*	0.96 (0.86, 1.07)	0.46
2. Potential confounders¶ and mediator variables§ - *'Direct’ effect*	1.01 (0.90, 1.13)	0.87
3. Unadjusted	0.95 (0.85, 1.06)	0.38

* Weights were determined as the median score for that morbidity (from 1 to 100, 100 being most severe) scored by nine independent anesthesiologists who were otherwise not involved in this study (appendix S2).

¶ Potential confounders: age, gender, race, body mass index, smoking status, dialysis, and ethanol alcohol (ETOH).

§ Mediator variables: congestive heart failure, hypertension, vascular surgery dilatations, vascular heart disease, carotid surgery, carotid disease, stroke, atrial fibrillation, atrial flutter, ventricular tachycardia, ventricular fibrillation, junctional, and myocardial infarction.

# Odds ratio for a 5-unit increase in vitamin D concentration.

Our sensitivity analyses showed that using the common effect GEE model instead of the *a priori*-chosen average relative effect GEE model would not have substantially changed results, and neither would ignoring the clinical severity weights. When ignoring severity weights the average relative effect GEE odds ratio (95% CI) (“total” effect) was 0.95 (0.87, 1.05). The common effect GEE odds ratio (95% CI) for the “total” effect was 0.94 (0.87, 1.01) when including severity weights and 0.92 (0.86, 0.99) when not including severity weights.

In addition, we observed that the associations were heterogeneous among the 11 individual cardiac morbidities (Vitamin D concentration -by-outcome interaction, P<0.001). We thus reported the individual associations between vitamin D and specific cardiac morbidities. After Bonferonni correction, there was no significant association between vitamin D concentration and any individual cardiac morbidity (Table 2).

**Table 2:** The associations between serum vitamin D concentration and individual cardiac morbidities among 426 cardiac surgical patients.

Individual cardiac morbidity	Incidence (%)	OR* (99.55% CI)†	P†
Asystole	3.3	1.26 (0.96, 1.66)	0.02
Atrial arrhythmia	30.3	0.95 (0.84, 1.08)	0.27
Cardioversion	8.2	0.90 (0.69, 1.17)	0.26
ECMO	1.2	1.27 (0.88, 1.84)	0.07
Heart Block	8.2	1.02 (0.85, 1.23)	0.77
IABP	0.9	0.54 (0.23, 1.25)	0.04
Low cardiac output	24.7	0.88 (0.76, 1.02)	0.02
Open Chest	5.2	0.96 (0.73, 1.26)	0.68
Permanent Pacer	1.2	1.14 (0.72, 1.80)	0.42
Pulmonary Edema	12.9	0.92 (0.76, 1.12)	0.25
VT/VF	10.1	0.85 (0.66, 1.08)	0.06

IABP = Intra-aortic balloon pump, ECMO = Extra corporeal membrane oxygenator, VT/VF = Ventricular tachycardia/Ventricular fibrillation.

* Odds ratio for a 5-unit increase in vitamin D concentration, after adjusting for potential confounders: age, gender, race, body mass index, smoking status, dialysis, and ethanol alcohol (ETOH).

† A Bonferroni correction was used to adjust for multiple testing. Thus, the 99.55% CIs are presented, and the significance criterion for each individual outcome is P<0.0045 (i.e., 0.05/11). None of the individual cardiac morbidities thus met our *a priori* criteria for statistical significance.

### Secondary Results

Vitamin D concentration was not associated with any of the secondary outcomes: neurologic morbidity (P = 0.27), surgical (P = 0.26) or systemic infections (P = 0.58), 30-day mortality (P = 0.55), or length of initial ICU stay (P = 0.04, Table 3). The incidences of respiratory morbidity ranged from 1.2% for aspiration pneumonia to 77% for atelectasis (Appendix S3). We also observed that 65% of patients used vasopressors on day of surgery or postoperative day 1. The association between vitamin D and use of vasopressor was not statistically significant (OR: 0.95 (0.88, 1.03) for a 5-unit increase in vitamin D concentration; P = 0.22), after adjusting for the confounding variables.

**Table 3:** The associations between serum vitamin D concentration and secondary outcomes among 426 cardiac surgical patients.

Secondary outcome	Incidence (%)	OR* (99% CI)	P
Neurologic morbidity	1.9	0.82 (0.51, 1.31)	0.27
Surgical infection	4.9	0.88 (0.67, 1.17)	0.26
Systemic infection	11.7	0.97 (0.82, 1.14)	0.58
30-day mortality	1.4	0.89 (0.55, 1.46)	0.55
	**Median [Q1, Q3]**	**HR** * **(99% CI)**	
Initial ICU LOS (days)¶	4 [2], [8]	1.04 (0.99, 1.09)	0.04

ICU = Intensive care unit; LOS = length of stay.

* Odds ratio or hazard ratio for a 5-unit increase in vitamin D concentration, after adjusting for potential confounders: age, gender, race, body mass index, smoking status, dialysis, and ethanol alcohol (ETOH).

¶ There summary statistics were length of stay for discharged alive patients. Six patients died in ICU; those patients were analyzed as never being discharged alive by assigning a follow-up time one day longer than the longest observed discharged alive time.

## Discussion

Optimal concentrations of serum vitamin D for various populations have been discussed extensively in recent years, resulting in a wide range of suggested therapeutic concentrations. Currently the most commonly accepted definition of vitamin D deficiency is a 25 (OH) D concentration less than 20 ng/ml, with vitamin D insufficiency defined as 21 to 29 ng/ml [11]. Using these definitions, 75% of our patients having cardiac surgery were deficient or insufficient. This is unsurprising as it is consistent with previous reports and since previous epidemiological studies have implicated vitamin D as an important marker of cardiovascular disease [8], [11], [21], [22].

The pathogenesis and the effect of vitamin D deficiency on cardiovascular disease is unclear. Low serum vitamin D concentrations are associated with inflammation, increased arterial stiffness, and endothelial dysfunction in human blood vessels [22]. Vitamin D also seems to inhibit renin-angiotensin II synthesis [23]. Therefore, mice lacking vitamin D receptors have elevated production of renin and angiotensin II, leading to hypertension, cardiac hypertrophy and increased water intake [24]. However, there is also the possibility of reverse causation: patients with serious cardiac problems may stay indoors more than others because of limited physical activity, in turn diminishing sun exposure and lowering vitamin D concentrations. Consistent with this theory, previous studies demonstrate that physical activity is closely related to serum vitamin D concentrations [25].

The most common cardiac complications observed in our current study were atrial arrhythmias, low cardiac output, and pulmonary edema. The combined incidence of these combined complications was 63%, which is comparable to previously reported cardiac surgery studies [26], [27]. In our study low vitamin D concentrations were not associated with increased cardiovascular complications after cardiac surgery. There are multiple potential explanations for our results; first, and perhaps most likely, the difference of our results from previous epidemiological studies suggests the possibility that vitamin D effects are relatively small, at least compared with the intense physiological insult of cardiac surgery. Second, hemodilution during cardiopulmonary bypass decreases Vitamin D concentrations, although only for about 24 hours, which may not be a clinically important duration – even in the context of surgical insult [28]. Furthermore our average vitamin D measurement day is 5 days prior surgery, which may have an effect on the results also, but it is very unlikely that these patients have had treatment and corrected their vitamin D levels. Third, lack of association may still be related to the limited sample size, although our *post hoc* analysis suggests that we had sufficient power to observe a clinically important association should one truly exist. And lastly, Vitamin D concentrations in our study ranged between 4.3 and 69 ng/ml. Some newer studies suggest that therapeutic concentrations might have to be greater than 50 ng/ml to significantly improve outcome.

Our study is one of the first Vitamin D study in the perioperative patient population. A very recent study contrary to our findings has demonstrated significant U shaped association with vitamin D levels [29]. The difference may have resulted from multiple factors; patient populations, how the outcomes were classified and evaluated but more importantly from the bypass technique. We know that off the pump technique is more often used in Europe then USA, which have significant effect on vitamin D concentrations. Similarly another study in neonates couldn’t demonstrate difference in outcomes other than vasopressor usage [30]. We were unable to demonstrate any difference in vasopressor usage, which may be result of very different patient populations. The closest other comparison might thus be to studies in the critical care populations in whom the association between vitamin D and outcome remains controversial. McKinney et al., for example, demonstrated increased mortality and length of hospital stay in veterans with low concentrations of vitamin D [31]. However, this retrospective analysis had a fairly limited sample size (136 patients), included patients over a long period of time (10 years) and did not adjust for time or center effect. Another study also found a close association with low vitamin D concentrations and increased length of hospital stay and mortality in ICU patients [32]. In contrast, a retrospective ICU study in septic and trauma patients did not identify an association between vitamin D concentrations and outcomes [33]. More importantly, a recent randomized and blinded trial showed no improvement in cardiac dysfunction or left ventricular mass index in high-risk patients with chronic kidney disease who were randomized to vitamin D treatment or placebo [34].

Vitamin D has significant immune modulating and antimicrobial function. Vitamin D receptor is present on B-lymphocytes, T lymphocytes, and monocytes [35]. Vitamin D effects the production of antimicrobial peptides like cathelicidin and β defensin [35], [36], [37]. These peptides, in addition to having immune modulatory functions, act as a line of defence against bacterial and viral infections. Most of this information comes from in-vitro experiments, however there are also some clinical studies supporting these findings. The most important association was shown for vitamin D and tuberculosis; incidence and susceptibility to active tuberculosis was higher in vitamin D deficient patients [38]. There are several studies evaluating the role of vitamin D in animal sepsis models, demonstrating significant decrease in pro-inflammatory cytokines with increased Vitamin D concentrations [39]. On the other hand vitamin D supplementation to reduce the occurrence of seasonal influenza yielded inconclusive results [40]. Our results also did not demonstrate an association with increased infectious complications and vitamin D concentrations. It is currently unknown whether vitamin D is only a marker of severity of certain diseases or a prognostic or diagnostic marker.

We applied novel statistical methods which allowed us to avoid a common pitfall of studies involving a composite endpoint, particularly, that the results are can easily be driven by the component(s) of the composite having the highest frequency, and those components may in fact be clinically least important [18], [41]. We applied the average relative effect generalized estimating equation (GEE) method discussed by Mascha and Imrey [18] which first estimates a treatment effect (i.e., log-odds ratio) for each outcome component and then averages them, so that no single component can overwhelm the others. This is in sharp contrast to the more standard GEE method, which estimates a single “common effect” across the components [42] and is thus susceptible to being driven by those with highest incidence. We also applied clinical severity weights so that components that are more important clinically would receive more weight in the analysis, regardless of the treatment effect or the incidence. We decided a priori to use the average relative effect model and to include clinical severity weights. Our sensitivity analyses to the chosen methods showed little impact of the severity weights themselves, and some impact due to using the average relative effect over the common effect odds ratio. The average relative effect method was most appropriate here because the components ranged in incidence from 1.2% (ECMO) to 30.3% (atrial arrhythmia).

Any retrospective analysis, including ours, potentially suffers from selection bias and confounding that are normally largely prevented by randomization. We used multivariable analysis to adjust for differences in potential confounding factors – but this approach is effective only for known confounders. Our list of available confounders is presumably incomplete; similarly, we at best have a crude estimate for the magnitude of most potential confounding factors. The extent to which selection bias and confounding contribute to our conclusions remains unknown, but could well be substantial. And finally, only 426 patients had vitamin D concentrations recorded. This number provides adequate power for cardiac morbidities, which are relatively common after cardiac surgery; however, we have marginal or inadequate power for rare secondary outcomes like mortality (1.4%) or neurologic (1.9%) complications.

This is one of the first clinical studies evaluating the effect of vitamin D on postoperative complications and outcomes after cardiac surgery. The basis for our analysis was compelling laboratory and epidemiologic evidence that adequate vitamin D concentrations are critical to cardiovascular health and resistance to infection. After controlling for all available confounding factors, we nonetheless found no evidence that postoperative outcomes were associated with perioperative Vitamin D concentrations. Our results thus suggest that perioperative vitamin D concentrations are not a clinically important predictor of adverse outcomes in patients undergoing cardiac surgery, probably because the outcomes are overwhelmingly determined by other baseline and surgical factors.

## Supporting Information

Appendix S1 Definition.
(DOCX)

Appendix S2 Definition incidence and severity score of primary outcomes - Cardiac morbidity (N = 426).
(DOCX)

Appendix S3 Definition and incidence of secondary outcomes (N = 426).
(DOCX)

Appendix S4 Summary of baseline characteristics between cardiac surgical patients included and excluded in our study.
(DOCX)

Appendix S5 Baseline and intraoperative characteristics for cardiac surgical patients by quartiles of serum vitamin D concentration (N = 426).
(DOCX)
