# Supplementary material for: Low Vitamin D Concentration Is Not Associated with Increased Mortality and Morbidity after Cardiac Surgery
Source: PLoS One. 2013 May 28;8(5):e63831. doi: 10.1371/journal.pone.0063831 (PMC3665712; doi:10.1371/journal.pone.0063831)
Supplement: Appendix S1 — Definition. (DOCX) [file pone.0063831.s001.docx]

**Appendix S1. Definition**

| **Outcome** | **Definition** |
| --- | --- |
| Dialysis | Renal dialysis for kidney failure. Only includes peritoneal and hemodialysis. |
| Myocardial infarction | MI within the specified time frame prior to the date of surgery as documented on either the history and physical or on the catheterization report MI ≤ 1 week, MI>1 wk & <3 mo, MI 3 - 6 months, and MI > 6 months. |
| Diabetes | Insulin dependent diabetes mellitus. Patient must currently be on insulin to control diabetes. Non-insulin dependent diabetes mellitus. Controlled by oral hypoglycemics. Oral hypoglycemics include DiaBeta, Diabinese, Glucotrol, Micronase, Orinase, and Tolinase. Diabetes mellitus controlled by diet only (include borderline diabetics). |
| Cardio shock | Patients with cardiogenic shock documented in the progress notes. |
| Endocarditis | Preoperative endocarditis as documented in the progress notes. |
| Congestive heart failure | Documented history of CHF and/or one of the following: SOB on exertion, PND (paroxysmal nocturnal dyspnea), orthopnea, or peripheral edema. |
| COPD / Asthma | Clinical history of COPD or asthma which required medication. Include patients with emphysema. The most commonly used medications include, proventil, atrovent, and any other bronchodilator, inhaled steroid, or beta-adrenergic drug. |
| Hypertension | History of hypertension requiring medical therapy. Does not include patients coming to the operating room who have elevated pressures |
| Vascular surgery or dilatations | History of vascular intervention; ie. fem-pop bypasses/dilatation’s, renal artery bypasses/dilatation’s, and abdominal aortic aneurysms. |
| Vascular heart disease | Patient has had prior vascular surgery and/or history of claudication, angio/noninvasive proven PVD, peripheral ASO (peripheral atherosclerotic occlusion), Leriche Syndrome, etc. |
| Carotid surgery | History of previous carotid surgery. |
| Carotid disease | Patient has had prior carotid surgery or > 40% occlusion of either carotid as proven by angiography or carotid ultrasound. |
| Stroke | Documented history with or without residual deficit. |
| Dysrhythmias | Any arrhythmia’s not included below |
| Atrial fibrillation  Atrial flutter  Ventricular tachycardia  Ventricular fibrillation  Junctional Rhythm | Patient has documented history of any of these  arrhythmia’s pre-op. |
