# Supplementary material for: Low Vitamin D Concentration Is Not Associated with Increased Mortality and Morbidity after Cardiac Surgery
Source: PLoS One. 2013 May 28;8(5):e63831. doi: 10.1371/journal.pone.0063831 (PMC3665712; doi:10.1371/journal.pone.0063831)
Supplement: Appendix S2 — Definition incidence and severity score of primary outcomes - Cardiac morbidity (N = 426). (DOCX) [file pone.0063831.s002.docx]

**Appendix S2. Definition incidence and severity score of primary outcomes - Cardiac morbidity (N = 426)**

| **Primary Outcome** | **Definition** | **Incidence (%)** | **Median of severity score^*^** | **Severity weight^** |
| --- | --- | --- | --- | --- |
| Asystole | Any pause in heart rate>10s requiring treatment with CPR pacer / drugs. | 3.3 | 70 | 0.15 |
| ECMO | Patient has extra corporeal membrane oxygenator placed in ICU. | 1.2 | 75 | 0.17 |
| IABP | Patient has intra-aortic balloon pump placed in ICU. | 0.9 | 60 | 0.13 |
| Open Chest | An open chest in ICU whether unable to close in the OR or opened for cardiac massage or hemodynamic instability in ICU. Does not include open chests due to infection. | 5.2 | 50 | 0.11 |
| VT/VF | Any life threatening ventricular arrhythmia requiring treatment. Documented up to 3 occurrences. | 10.1 | 40 | 0.09 |
| Atrial arrhythmia | Atrial arrhythmia requiring treatment. Includes atrial fibrillation, atrial flutter, supraventricular tachycardia, and premature atrial contractions. | 30.3 | 20 | 0.04 |
| Cardioversion | DCC for A-Fib. Documented up to 3 occurrences. | 8.2 | 30 | 0.07 |
| Heart Block | Nodal rhythm, new bundle branch block, complete heart block as documented per cardiology progress notes. Includes junctional rhythm, and first and second-degree heart blocks. | 8.2 | 20 | 0.04 |
| Low cardiac output | Cardiac index < 1.8 liters/min/m2 despite adequate fluid replacement and on high dose inotropes for > 4 hrs. The CO should remain < 1.8 for majority of the 4 hrs. Include all patients on ventricular assist devices. | 24.7 | 40 | 0.09 |
| Permanent Pacer | Anytime a patient is sent to the pacer lab for insertion of a permanent pacer. The patient may return to the CVICU, the floors, or CICU directly from the pacer lab. | 1.2 | 20 | 0.04 |
| Pulmonary Oedema | Documented in progress notes or by chest x-ray report; symptoms include pink frothy sputum from endotracheal tube, elevated CVP, and Pulmonary pressures (if a PA catheter is available). Patient also presents with rales and/or rhonchi upon chest auscultation. | 12.9 | 30 | 0.07 |

IABP = Intra-aortic balloon pump, ECMO = Extra corporeal membrane oxygenator, VT/VF = Ventricular tachycardia / fibrillation, PA =pulmonary artery, CVICU = cardiovascular intensive care unit, CICU = coronary intensive care unit, CVP = central venous pressure, CO = cardiac output, ICU = intensive care unit, CPR = cardiopulmonary resuscitation, OR = operating room

* 1 to 100, 100 being most severe; evaluated by 9 independent anaesthesiologists who were otherwise uninvolved in this study.
^ severity weights (based on median severity scores) across the components forced to sum to 1.0 so that the average relative effect odds ratio is an average of the individual log-odds ratios[[18](#_ENREF_18)]
