# Supplementary material for: Low Vitamin D Concentration Is Not Associated with Increased Mortality and Morbidity after Cardiac Surgery
Source: PLoS One. 2013 May 28;8(5):e63831. doi: 10.1371/journal.pone.0063831 (PMC3665712; doi:10.1371/journal.pone.0063831)
Supplement: Appendix S3 — Definition and incidence of secondary outcomes (N = 426). (DOCX) [file pone.0063831.s003.docx]

**Appendix S3. Definition and incidence of secondary outcomes (N = 426)**

| **Secondary Outcome** | **Definition** | **Incidence (%)** |
| --- | --- | --- |
| **Mortality** | All-cause 30-day mortality. | 1.4 |
| **Neurologic morbidity** | | |
| Focal Deficit | Symptoms related to focal central lesion (aphasia, decrease in lymph function or hemiparesis); may or may not have documented central lesion by CT scan. This is a symptomatic description. | 1.9 |
| Global Deficit | Symptoms global in nature (i.e. watershed infarcts). Patients generally do not regain consciousness after the event. Patients regaining consciousness will gradually deteriorate. Documented by symptomatology, CT scan, or electrical encephalogram (EEG). | 0 |
| **Surgical infections** | | |
| Empyema | Collection of purulent material in the pleural space. Positive diagnosis occurs when a chest tube is inserted in the pleural space with positive cultures grown from the chest tube drainage. | 0.7 |
| Endocarditis | Developed postoperatively. Fever should be > 38 C. Requires an organism isolated from the culture of the valve or vegetation, or two of the following with no recognized cause: 1) Positive culture on gram stain. 2) Valve vegetation’s seen during valve surgery. 3) Evidence of new vegetation on echocardiogram. | 1.2 |
| Mediastinitis | Sternal click, open sternal wound, drainage from the mediastinal incision, with elevated temperature. Sternal click alone does not qualify. Patient is returned to the operating room with operative note diagnosing mediastinitis or sternectomy with muscle flap grafts to the affected area. Diagnosis should include organism isolated from the cultures along with elevated temperature, elevation of WBCs and the institution of antimicrobial therapy, and possibly Betadine irrigation of the area. | 0.9 |
| Sternal Wound infection | Sternal wound infection other than Mediastinitis, documented with positive cultures, requiring intervention. | 2.1 |
| Wound | Excludes mediastinal wounds. Purulent drainage with positive cultures and institution of therapy. Wound may dehiss or be opened by surgeons. | 1.4 |
| **Systemic infections** | | |
| Bacteremia | Bacteria grown from blood cultures which are treated with appropriate therapy. Must be documented by Infectious Disease. | 8.2 |
| Fungemia | Patient has a fungal infection which is treated with an antifungal medication. Documented by Infectious Disease. | 0.5 |
| Line Sepsis | Documented by Infectious Disease or ICU team in the progress notes along with positive line culture tips. | 2.6 |
| Sepsis Syndrome | Sepsis with evidence of altered organ perfusion. Must be documented by Infectious Disease or the ICU service. It may be expressed as tachycardia, fever or hypothermia, tachypnea, and evidence of inadequate organ perfusion. | 4.9 |
| Septic Shock | A sepsis syndrome characterized by hypotension (systolic BP < 90 mmHg or decrease from baseline > 40 mmHg). Patient should have increased cardiac output. BP is responsive to fluids and drugs. | 1.9 |
| **Respiratory morbidity** | | |
| Pneumonia | Patient presents with fever>38°C, elevation in WBCs, increase in sputum production, infiltrate on chest x-ray which does not clear in 24 hrs and has positive sputum culture. Documented by Infectious Disease. | 6.1 |
| ARDS | Documented by I.D. / ICU team, chest x-ray. Acute onset with clinical diagnosis of PaO_2_ < 60 mmHg, significant infiltrate on x-ray which is consistent with diagnosis, decreased lung compliance < 0.5 ml/cm H_2_O. | 1.4 |
| Aspiration pneumonia | Clinical diagnosis requires a documented episode of aspiration and infiltrate on chest x-ray consistent with diagnosis. | 1.2 |
| Atelectasis | Significant atelectasis documented in progress notes or by chest x-ray with institution of appropriate therapy of intermittent positive pressure breathing, incentive spirometry, or bronchoscopy for removal of a mucus plug. | 77.0 |
| Bronchospasms | Documented in the progress notes. Patient presents with wheezing requiring treatment with a bronchodilator. | 10.6 |
| Respiratory Insufficient/distress | Patient requires one or more of the following: FiO_2_≥80% for ≥24 hours or Mask CPAP | 11.0 |
| Respiratory failure | Patient's PaCO2 is > 50 mmHg and patient requires reintubation. | 6.1 |
| **Use of vasopressor** | On either day of surgery or postoperative day 1 | 65.7 |

ARDS = Acute respiratory distress syndrome, CPAP = continuous positive airway pressure, PaCO2 = partial pressure of arterial carbon dioxide, PaO2 = partial pressure of arterial oxygen, ICU = intensive care unit, WBC = white blood cells, BP = blood pressure, CT = computed tomography, EEG = electroencephalogram
