# Supplementary material for: Low Vitamin D Concentration Is Not Associated with Increased Mortality and Morbidity after Cardiac Surgery
Source: PLoS One. 2013 May 28;8(5):e63831. doi: 10.1371/journal.pone.0063831 (PMC3665712; doi:10.1371/journal.pone.0063831)
Supplement: Appendix S4 — Summary of baseline characteristics between cardiac surgical patients included and excluded in our study. (DOCX) [file pone.0063831.s004.docx]

| **Appendix S4. Summary of baseline characteristics between cardiac surgical patients included and excluded in our study** | | | |
| --- | --- | --- | --- |
| **Variable** | **Included**  **(N = 426)** | **Excluded**  **(N = 17,638)** | **SD** |
| Age, yr | 60 ± 14 | 63 ± 14 | -0.21 |
| Gender (female), % | 32 | 34 | -0.05 |
| Race (Caucasian), % | 83 | 90 | -0.19 |
| Body mass index, kg/m^2^ | 27 [23, 31] | 28 [24, 32] | -0.12 |
| Smoking (current / ever), % | 52 | 50 | 0.05 |
| ETOH ^§^, % | 9 | 11 | -0.07 |
| Dialysis, % | 6 | 2 | 0.19 |
| ASA status (IV vs. III), % | 94 | 80 | 0. 44 |
| Hematocrit, % | 36 ± 6 | 40 ± 6 | -0.77 |
| Blood urea nitrogen, mg/dL | 24 [17, 36] | 18 [15, 24] | 0.51 |
| Creatinine, mg/dL | 1.2 [1.0, 1.7] | 1.0 [0.8, 1.2] | 0.57 |
| Albumin, U/L | 3.8 [3.3, 4.2] | 4.2 [3.8, 4.5] | -0.65 |
| Bilirubin, mg/dL | 0.7 [0.5, 1.1] | 0.6 [0.4, 0.8] | 0.33 |
| Myocardial infarction, % | 34 | 22 | 0.26 |
| Diabetes, % | 34 | 24 | 0.22 |
| Cardio shock, % | 10 | 1 | 0.40 |
| Endocarditis, % | 2 | 3 | -0.06 |
| Congestive heart failure, % | 69 | 27 | 0.93 |
| COPD / Asthma, % | 15 | 12 | 0.12 |
| Hypertension, % | 63 | 68 | -0.12 |
| Vascular surgery dilatations, % | 8 | 5 | 0.13 |
| Vascular heart disease, % | 17 | 11 | 0.17 |
| Carotid surgery, % | 4 | 4 | -0.00 |
| Carotid disease, % | 15 | 15 | 0.03 |
| Stroke, % | 11 | 8 | 0.10 |
| Dysrhythmias, % | 34 | 14 | 0.48 |
| Atrial fibrillation, % | 34 | 23 | 0.24 |
| Atrial flutter, % | 2 | 1 | 0.03 |
| Ventricular tachycardia, % | 15 | 2 | 0.46 |
| Ventricular fibrillation, % | 3 | 1 | 0.18 |
| Junctional rhythmus, % | 0 | 0 | 0.01 |
| ASA = American Society of Anesthesiologists; COPD = Chronic obstructive pulmonary disease  ^§^ 1drink/day or > 3 drinks/wk  Summary statistics are presented as % of patients, mean ± SD, or median [Q1, Q3], respectively. | | | |
