# Supplementary material for: Low Vitamin D Concentration Is Not Associated with Increased Mortality and Morbidity after Cardiac Surgery
Source: PLoS One. 2013 May 28;8(5):e63831. doi: 10.1371/journal.pone.0063831 (PMC3665712; doi:10.1371/journal.pone.0063831)
Supplement: Appendix S5 — Baseline and intraoperative characteristics for cardiac surgical patients by quartiles of serum vitamin D concentration (N = 426). (DOCX) [file pone.0063831.s005.docx]

**Appendix S5. Baseline and intraoperative characteristics for cardiac surgical patients by quartiles of serum vitamin D concentration (N = 426)**

| **Variable** | **Serum Vitamin D Concentration (ng/mL)** | | | | **P ^a^** |
| --- | --- | --- | --- | --- | --- |
|  | **< 12.5**  **(N = 108)** | **12.5 – 19**  **(N = 104)** | **19 – 30**  **(N = 108)** | **≥ 30**  **(N = 106)** |  |
| Age, yr | 59 ± 14 | 57 ± 14 | 62 ± 13 | 61 ± 14 | 0.03 ^b^ |
| Gender (male), % | 66 | 72 | 72 | 63 | 0.38 |
| Race (Caucasian), % | 73 | 82 | 93 | 86 | 0.001 |
| Body mass index | 28 [24, 32] | 27 [23, 32] | 27 [23, 31] | 26 [24, 30] | 0.27 ^c^ |
| Smoking (current / ever), % | 53 | 42 | 60 | 54 | 0.07 |
| ETOH ^§^, % | 6 | 9 | 6 | 17 | 0.01 |
| Dialysis, % | 8 | 8 | 4 | 4 | 0.31 |
| ASA status (IV vs. III), % | 95 | 91 | 97 | 92 | 0.19 |
| Myocardial infarction, % | 38 | 36 | 40 | 23 | 0.04 |
| Diabetes, % | 40 | 28 | 38 | 28 | 0.13 |
| Cardio shock, % | 13 | 10 | 10 | 8 | 0.74 |
| Endocarditis, % | 0 | 2 | 1 | 5 | 0.06 |
| Hematocrit, % | 34 ± 6 | 36 ± 6 | 36 ± 6 | 36 ± 6 | 0.05 ^b^ |
| Blood urea nitrogen, mg/dL | 24 [17, 40] | 21 [17, 28] | 26 [20, 36] | 24 [17, 39] | 0.11 ^c^ |
| Creatinine, quantile mg/dL | 1.2 [1.0, 1.8] | 1.2 [1.0, 1.7] | 1.3 [1.0, 1.6] | 1.3 [1.0, 1.7] | 0.43 ^c^ |
| Albumin, U/L | 3.5 [3.0, 3.9] | 3.8 [3.4, 4.2] ^*^ | 3.7 [3.2, 4.2] ^*^ | 4.0 [3.5, 4.3] | <.001 ^c^ |
| Bilirubin, mg/dL | 0.7 [0.4, 1.0] | 0.7 [0.5, 1.3] ^*^ | 0.7 [0.4, 1.1] ^*^ | 0.7 [0.5, 1.1] | 0.88 ^c^ |
| *Medical history* |  |  |  |  |  |
| Congestive heart failure, % | 78 | 66 | 75 | 57 | 0.003 |
| COPD / Asthma, % | 19 | 13 | 18 | 12 | 0.51 |
| Hypertension, % | 66 | 61 | 61 | 63 | 0.86 |
| Vascular surgery dilatations, % | 12 | 7 | 6 | 8 | 0.33 |
| Vascular heart disease, % | 22 | 12 | 16 | 17 | 0.29 |
| Carotid surgery, % | 6 | 2 | 5 | 4 | 0.58 |
| Carotid disease, % | 21 | 8 | 19 | 13 | 0.03 |
| Stroke, % | 16 | 11 | 8 | 8 | 0.20 |
| Dysrhythmias, % | 31 | 30 | 40 | 33 | 0.43 |
| Atrial fibrillation, % | 31 | 30 | 38 | 37 | 0.47 |
| Atrial flutter, % | 2 | 2 | 3 | 1 | 0.81 |
| Ventricular tachycardia, % | 19 | 12 | 19 | 7 | 0.02 |
| Ventricular fibrillation, % | 6 | 2 | 2 | 3 | 0.18 |
| Junctional Rhythm, % | 0 | 0 | 0 | 0 | 0.99 |
| *Intraoperative* |  |  |  |  |  |
| Aortic valve replace, % | 20 | 12 | 19 | 22 | 0.32 |
| Aortic valve repair, % | 1 | 0 | 1 | 1 | 0.81 |
| Mechanical valve replace, % | 9 | 5 | 8 | 7 | 0.61 |
| Mechanical valve repair, % | 15 | 10 | 14 | 11 | 0.65 |
| Tissue valve replace/repair, % | 18 | 13 | 12 | 8 | 0.26 |
| Duration of surgery, hrs | 7 [6, 8] | 7 [6, 8] | 7 [6, 8] | 6 [6, 8] | 0.65 ^c^ |

ASA = American Society of Anaesthesiologists; COPD = Chronic obstructive pulmonary disease, ETOH = ethanol alcohol

^§^ 1drink/day or > 3 drinks/week

Summary statistics are presented as % of patients, mean ± SD, or median [Q1, Q3], respectively.

^*^ 1 patient had a missing value.

^a^ Pearson's chi-squared test, unless specified; ^b^ ANOVA; and ^c^ Kruskal-Wallis ANOVA by ranks.
